# Annual acknowledgement of manuscript reviewers

**DOI:** 10.1186/1748-5908-9-9

**Published:** 2014-01-21

**Authors:** Anne Sales, Michel Wensing

**Affiliations:** 1 Co-Editors-in-Chief, Implementation Science

## Abstract

**Contributing reviewers:**

The editors of Implementation Science would like to thank all our reviewers who have contributed to the journal in Volume 8 (2013).

## 

Charles Abraham

United Kingdom

Sara Ackerman

United States of America

Maria Ahmed

United Kingdom

Lauren Albrecht

Canada

Marlyn Allicock

United States of America

Albert Alonso

Spain

Felicity Astin

United Kingdom

Lou Atkins

United Kingdom

Neal Axon

United States of America

Ross Bailie

Australia

Emily Bain

Australia

Mark Baker

United Kingdom

Luciana Ballini

Italy

Michelle Banfield

Australia

Chris Barker

United Kingdom

Melba Andrea Baylon

Canada

Monika Becker

Germany

Hilary Bekker

United Kingdom

Jonathan Benn

United Kingdom

Sally Bennett

Australia

Guillermo Bernal

United States of America

Fiona Beyer

United Kingdom

Tom Blakeman

United Kingdom

Gary Bond

United States of America

Andrew Booth

United Kingdom

Jennifer Boyko

Canada

Anke Bramesfeld

Germany

Melissa Brouwers

Canada

C. Hendricks Brown

United States of America

Jako Burgers

Netherlands

Richard Byng

United Kingdom

Suzanne Cadarette

Canada

Judith Callan

United States of America

Simon Capewell

United Kingdom

Stacy Carter

Australia

Martin Cartwright

United Kingdom

Raquel Castillo-Oyagüe

Spain

Enrique Castro-Sánchez

United Kingdom

David Chambers

United States of America

Esmita Charani

United Kingdom

Charles Collins

United States of America

Joanne Collins

Australia

Heather Colquhoun

Canada

Julie Considine

Australia

Geoffrey Curran

United States of America

Janet Curran

Canada

Gavin Daker-White

United Kingdom

Laura Damschroder

United States of America

Barbara Davies

Canada

Huw Davies

United Kingdom

Rachel Davis

United Kingdom

Martin Dawes

Canada

Carl de Wet

United Kingdom

Christopher Deery

United Kingdom

Sara Demain

United Kingdom

Sarah Dennis

Australia

Marcel Dijkers

United States of America

Carmen Dirksen

Netherlands

Diane Dixon

United Kingdom

Patricia Documet

United States of America

Peggy Dolcini

United States of America

Shannon Dorsey

United States of America

Dawn Dowding

United Kingdom

George Dowswell

United Kingdom

Lydia Drumright

United Kingdom

Joseph Durlak

United States of America

Judith Dyson

United Kingdom

Anne G. Ekeland

Norway

Glyn Elwyn

United Kingdom

Cecile Emery

United Kingdom

Mike English

Kenya

Catrin Evans

United Kingdom

Ken Farrington

United Kingdom

Ewan Ferlie

United Kingdom

Caroline Finch

Australia

Tracy Finch

United Kingdom

Sarah Flicker

Canada

Ivan Dario Florez

Colombia

Jane Forman

United States of America

Jill Francis

United Kingdom

Tobias Freund

Germany

John Gabbay

United Kingdom

Anna Gagliardi

Canada

Marie-Pierre Gagnon

Canada

Heather L. Gainforth

Canada

Francois-Pierre Gauvin

Canada

Anik Giguere

Canada

Justin Glasgow

United States of America

Russell E. Glasgow

United States of America

Elliot Michael Goldner

Canada

Ralph Gonzales

United States of America

David Goodrich

United States of America

Ian Graham

Canada

Gonzalo Grandes

Spain

Carla A. Green

United States of America

Kelly Grindrod

Canada

Peter Groenewegen

Netherlands

Julie Hadley

United Kingdom

Martin Hagger

Australia

Michael Hall

United States of America

Alison Hamilton

United States of America

Stephen Robert Hanney

United Kingdom

Wendy Hardeman

United Kingdom

Janet Harris

United Kingdom

Margaret B. Harrison

Canada

Molly Harrod

United States of America

Katharina Hauck

United Kingdom

Donna Havens

United States of America

Emma Healey

United Kingdom

Jacqueline Hebert

Canada

Naomi Heijmans

Netherlands

Christian Helfrich

United States of America

Susanne Hempel

United States of America

Amanda Henderson

Australia

Anne Hickey

Ireland

Pat Hoddinott

United Kingdom

William Hogg

Canada

Daniel Holt

United States of America

Alison Hutchinson

Australia

Sylvia Hysong

United States of America

Irene Ilott

United Kingdom

David Inwald

United Kingdom

Theodore Iwashyna

United States of America

Jesse Jacobs

United States of America

Justin Jagosh

Canada

Rozh Jalil

United Kingdom

Jalila Jbilou

Canada

Lianne Jeffs

Canada

Ray Jones

United Kingdom

Monika Kastner

Canada

Elizabeth Kendall

Australia

Amy Kilbourne

United States of America

Roman Kislov

United Kingdom

Alison Kitson

Australia

Jonathan Klein

United Kingdom

Jan Koetsenruijter

Netherlands

Gerjo Kok

Netherlands

Flora Kokkinaki

Greece

Niina Kolehmainen

United Kingdom

Anita Kothari

Canada

Sarah Krein

United States of America

Yiannis Kyratsis

United Kingdom

Benjamin Lamb

United Kingdom

Sue Latter

United Kingdom

Anna Lau

United States of America

Rachel Laws

Australia

Rebecca Lawton

United Kingdom

Julie Leask

Australia

Martin Lee

United States of America

Jennifer Leeman

United States of America

France Légaré

Canada

Lori Letts

Canada

Laura Leviton

United States of America

William Livingood

United States of America

Paul Lord

United Kingdom

Fabiana Lorencatto

United Kingdom

Meryl Lovarini

Australia

Julie Lowery

United States of America

Carl Macrae

United Kingdom

Kamal Ram Mahtani

United Kingdom

Reza Majdzadeh

Iran

David Mandell

United States of America

Barbara Mark

United States of America

Martin Marshall

United Kingdom

Steve Martino

United States of America

Margaret Maxwell

United Kingdom

Carl May

United Kingdom

Danielle Mazza

Australia

John McAteer

United Kingdom

Catriona McDaid

United Kingdom

Rosemary McEachan

United Kingdom

Bradford McFadyen

Canada

Ann McKibbon

Canada

Sandy Middleton

Australia

Rhona Mijumbi

Uganda

Gabriel Moore

Australia

Tim Moore

Australia

Chris Morris

United Kingdom

Rebecca Morris

United Kingdom

Alastair Munro

United Kingdom

Mary Ann Murray

Canada

Laura Nichols

United States of America

Paul Norman

United Kingdom

Christine Norton

United Kingdom

Wynne Norton

United States of America

Jacinta Nzinga

Kenya

Eivor Oborn

United Kingdom

Alicia O'Cathain

United Kingdom

Kathryn Oliver

United Kingdom

Carola Orrego

Spain

Lois Orton

United Kingdom

Richard Osborne

Australia

Matthew Page

Australia

Lawrence Palinkas

United States of America

Julie Parkes

United Kingdom

Elena Parmelli

Italy

Alan Pearson

Australia

Jeremy Pearson

United Kingdom

Frank Peters-Klimm

Germany

K. V. Petrides

United Kingdom

Lourdes Planas

United States of America

Constance Dimity Pond

Australia

Michael Potter

United States of America

Justin Presseau

United Kingdom

Enola Proctor

United States of America

Audrey Prost

United Kingdom

Andrew Quanbeck

United States of America

Padmanabhan Ramnarayan

United Kingdom

Craig Ramsay

United Kingdom

Tim Rapley

United Kingdom

Gwyneth Rees

Australia

Michael Rigby

Ireland

Erin Riley

Australia

Kerstin Roback

Sweden

Scott Roesch

United States of America

Rasa Ruseckaite

Australia

Stephanie Russ

United Kingdom

Jo Rycroft-Malone

United Kingdom

Anna Sallis

United Kingdom

Jane Sandall

United Kingdom

Caroline Sanders

United Kingdom

Isabelle Scholl

Germany

Shannon Scott

Canada

Luca Dan Serbanati

Italy

Rod Sheaff

United Kingdom

Donna Shelley

United States of America

Camille Short

Australia

Kathryn Sibley

Canada

Jane Silovsky

United States of America

Francis Silvestri

United States of America

Julius Sim

United Kingdom

Laura Siminoff

United States of America

Lesli Skolarus

United States of America

Karen Smith

United Kingdom

Samuel Smith

United Kingdom

Karla Soares-Weiser

United Kingdom

Sophie Soklaridis

Canada

Nina Sperber

United States of America

Mandy Stanley

Australia

Nicholas Steel

United Kingdom

Ian Steen

United Kingdom

Tim Stokes

United Kingdom

Shannon Stokley

United States of America

Julie Storr

United Kingdom

Mathilde Strating

Netherlands

Angela Sweeney

United Kingdom

Gregory Teague

United States of America

Victoria Thomas

United Kingdom

Richard Thomson

United Kingdom

Kevin Thorpe

Canada

Lyndal Trevena

Australia

Linda Trinh

Canada

Tari Turner

Australia

Sara Twaddle

United Kingdom

Thomas Valente

United States of America

Pieter van den Hombergh

Netherlands

Henk F. van der Molen

Netherlands

Trudy van der Weijden

Netherlands

Andrei Vasilateanu

Romania

Thomas Vaughn

United States of America

Edward Velasco

Germany

Theo Verheij

Netherlands

Wim V. Verstappen

Netherlands

Charles Vincent

United Kingdom

Ivo Vlaev

United Kingdom

Lars Wallin

Sweden

Abe Wandersman

United States of America

Vicky Ward

United Kingdom

Robert Wears

United States of America

Jan-Willem Weenink

Netherlands

Bryan Weiner

United States of America

Kenneth Wells

United States of America

Gill Westhorp

Australia

John Wiggers

Australia

Brian Williams

United Kingdom

Paul Wilson

United Kingdom

Shannon Wiltsey Stirman

United States of America

Peter Wyer

United States of America

Naohito Yamaguchi

Japan

Ian Young

United Kingdom

Jane Young

Australia

